# Ultrasound-guided peripheral venous cannulation in critically ill patients: a practical guideline

**DOI:** 10.1186/s13089-019-0144-5

**Published:** 2019-10-17

**Authors:** Pablo Blanco

**Affiliations:** Intensive Care Unit, Clínica Cruz Azul, 2651, 60 St, 7630 Necochea, Argentina

**Keywords:** Ultrasound, Point-of-care, Peripheral venous catheterization

## Abstract

**Background:**

Up to one-third of critically ill patients have difficult intravenous access (DIVA). This occurs often in obese patients, those with generalized edemas or in patients with previous venous cannulations. In DIVA patients, the conventional technique often fails. In contrast, ultrasound-guided cannulation has demonstrated a high success rate, improving patient satisfaction and even a reduction in the need of central venous lines. However, a high rate of premature catheter failure has been shown in cannulations performed by ultrasound guidance and thus a comprehensive knowledge of several aspects related to this procedure is mandatory to improve cannulation success, avoid complications and lengthen the survival of the catheter.

**Main text:**

Several practical issues related to peripheral venous cannulation are described: peripheral venous anatomy, vein size and catheter selection, distance from skin to vein, insertion angle and selection of the catheter length, cannulation technique itself (out-of-plane or in-plane) and checking catheter position.

**Conclusion:**

Key concepts regarding ultrasound-guided peripheral vein cannulation should be well known for practitioners, aiding in improving cannulation success and catheter dwell time, and avoiding complications.

## Background

**Fig. 1:**
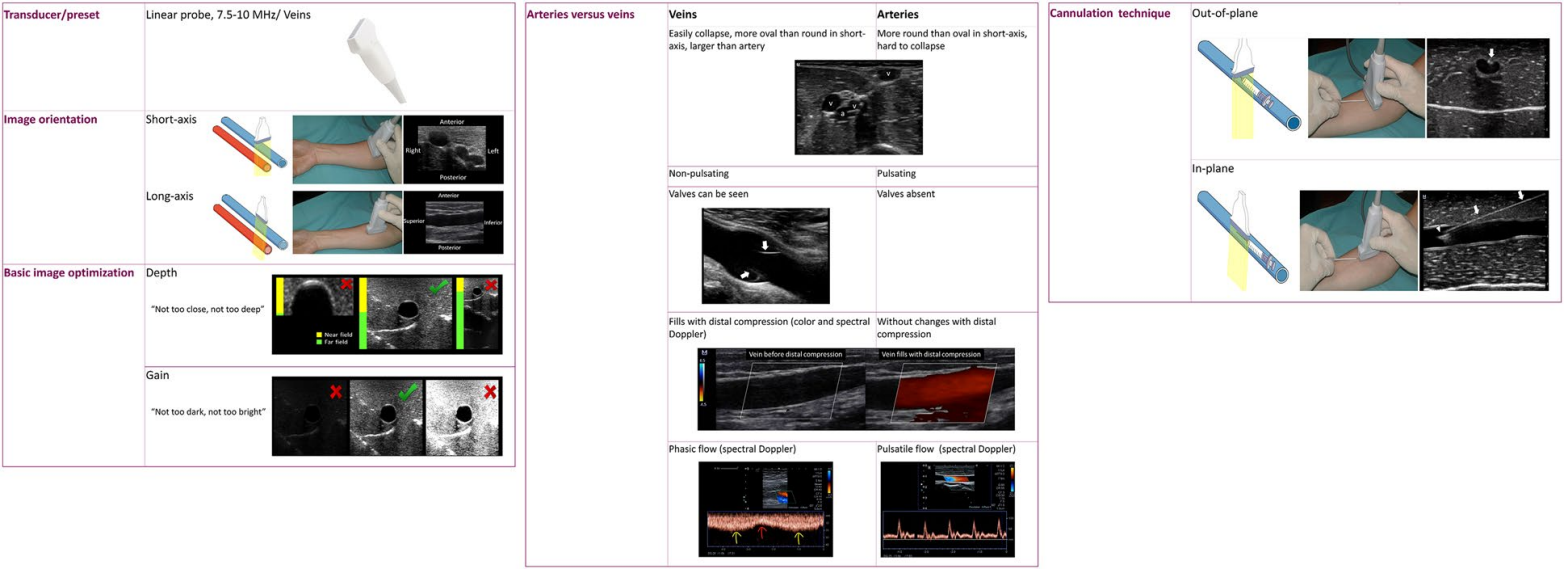
Basic general knowledge for ultrasound-guided vascular cannulation: selection of the transducer and preset, image orientation, basic image optimization, distinguishing veins versus arteries and the two techniques of cannulation used in practice: the out-of-plane and the in-plane technique

Peripheral venous cannulation is essential to provide care for the patients in the emergency department or critical care unit. While most intravenous catheters are placed using the conventional technique (i.e., seeing and/or palpating the vein), up to one-third of the patients have difficult intravenous access (DIVA) [1]. This group often involves patients with generalized edemas, obese, those with multiple previous cannulations or intravenous drug users [2–5]. For patients who have DIVA, ultrasound (US)-guided cannulation has shown an overall success rate higher than 90%, compared to 25–30% using the conventional technique [2–4, 6], and also aids in reducing the need for central venous lines [4, 5]. Patient satisfaction also improves using US guidance [2]. In spite of that, the rate of premature catheter failure (PCF), which may account for as high as 50% within 24 h of catheter placement, is higher with US-guided cannulations (45–56%) compared to the conventional technique (19–25%) [1, 6, 7]. Infiltration is the leading cause of catheter failure; catheter dislodgement and thrombophlebitis are also common [1, 6]. Key concepts regarding veins, catheters and the technique itself should be considered by practitioners to improve success, reduce complications and improve dwell times in US-guided peripheral intravenous cannulation, and these are provided in this article, and are summarized in Table 1 as well. Basic general knowledge for US-guided vascular cannulation is shown in Fig. 1.

**Table 1:** Key concepts for ultrasound-guided peripheral venous cannulation

Key concept	Description	Considerations
Must know	Basic general knowledge for ultrasound-guided vascular cannulation	Selection of the transducer and preset; image orientation; basic image optimization; distinguishing veins versus arteries; managing techniques of cannulation (out-of-plane and in-plane technique)
1	Select superficial veins (i.e., epifascial)	Superficial veins: short pathway to reach the vein; high probability that a great proportion of the catheter will dwell in the vein Deep veins: inherent risks of needle-stick injury of the artery or the nerve; frequent catheter dislodgment
2	Patent veins	Anechoic lumen; fully compressible Do not misinterpret stagnant blood in the vein lumen with thrombus (distal compression is useful)
3	Vein size: anteroposterior diameter ≥ 4 mm	AP diameter (mm) = maximum Fr catheter size (e.g., 4 mm = up to 4-Fr catheter)
4	Vein depth: up to 16 mm (short axis)	Real distance to reach the vein (45° insertion angle)*: 1.4 × vertical distance *Ideally measure real distance in long axis and select the best angle of insertion ≥ 2.75 cm of the catheter must dwell in the vein Consider using ultra-long peripheral (6.3 cm) and midline (8–20 cm) catheters to minimize catheter failure
5	Select the technique: in-plane or out-of-plane technique	Both are useful, although it seems to be a greater success rate with the out-of-plane technique Learning and using both techniques is encouraged
6	Checking catheter position	Direct: double hyperechoic line into the lumen vein Indirect: saline flush test (two-dimensional and/or color Doppler)

### Key concept 1: Select superficial veins

**Fig. 2:**
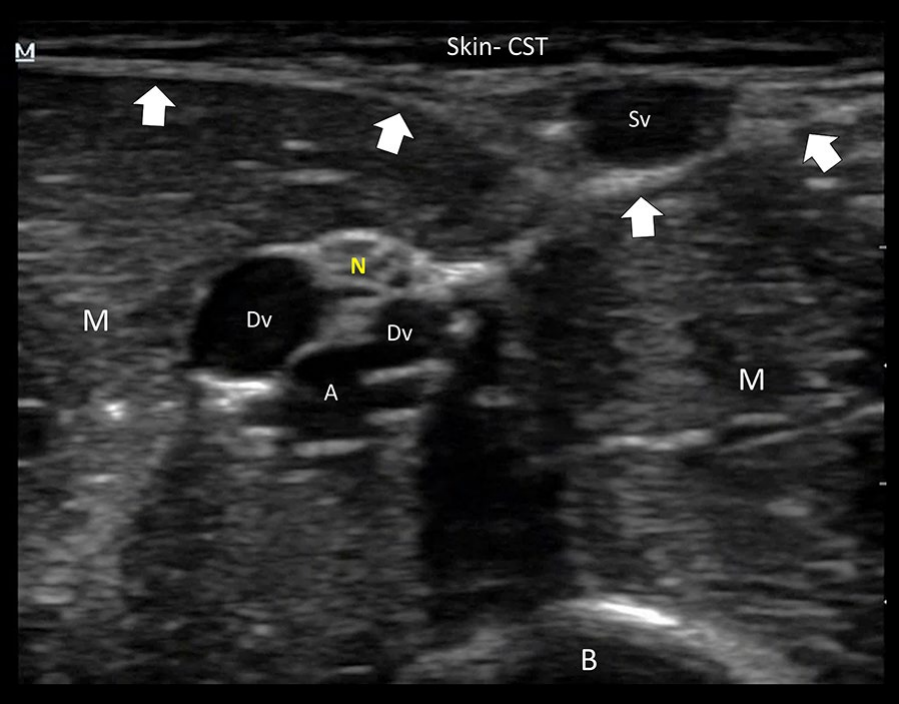
Ultrasound anatomy applied to cannulation of the peripheral veins of the upper limbs. Superficial veins (Sv) are found above fascia (delineated by arrows) in the cellular subcutaneous tissue (CST), and deep veins below fascia, or subfascial. As noted, deep veins are paired (brachial veins in this case), and are accompanied by an artery (A, brachial artery in this example) and a nerve (N, median nerve in this case) in the neurovascular bundle. The muscle (M) and the bone (B) are also found in the subfascial compartment. Superficial veins should be selected for cannulation

**Fig. 3:**
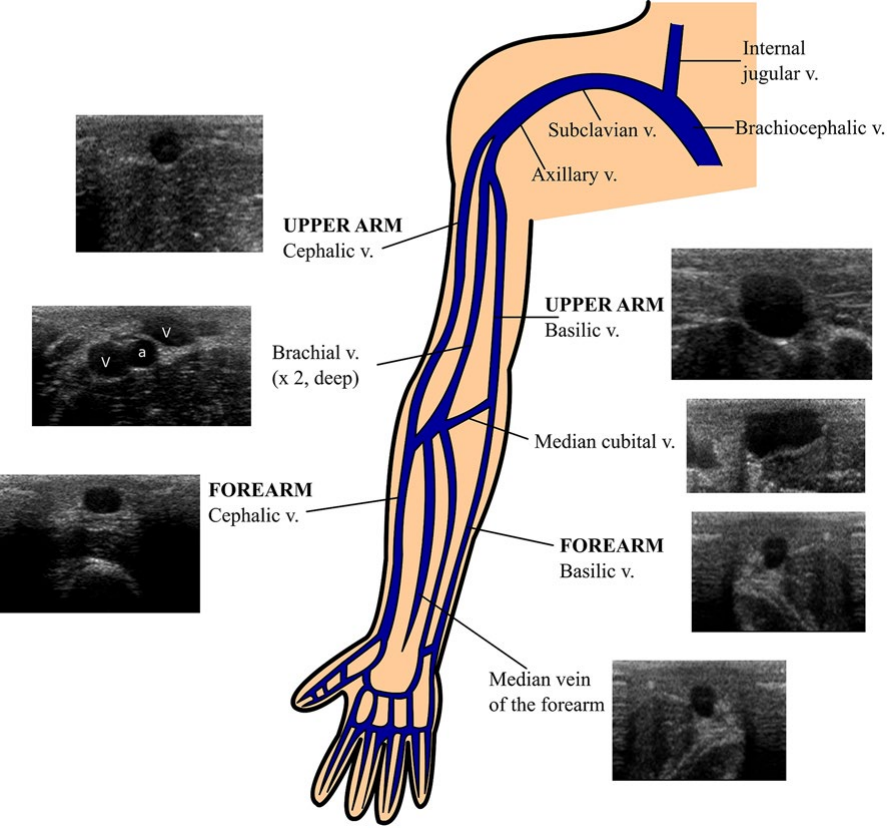
Anatomy of the peripheral veins of the upper limbs. *v* vein

Anatomically, peripheral veins may be superficial or epifascial (i.e., above the fascia), and deep or subfascial (i.e., below the fascia). Superficial veins are found close to the skin and travel without an accompanying artery or nerve. In contrast, deep veins (which may be paired, as seen in brachial veins) are found at the neurovascular bundle, and thus are accompanied by an artery and a nerve (Fig. 2 and Additional file 1: Video S1). From these anatomical points, some key concepts advocate for the use of superficial veins instead of deep veins. Using superficial veins provides a short pathway to cannulation, leads to dwell a higher proportion of the catheter inside the vein (an issue intimately related to PCF, see below) and as a safe issue, avoids needle-stick injury of the artery or nerve. Cannulation of deep veins is also associated with a greater risk of catheter dislodgment when compared to cannulation of superficial veins [3, 8]. The superficial veins of the upper limbs which can be cannulated are the basilic (found medial, in the upper arm and in the forearm), and the cephalic (found lateral, in the upper arm and in the forearm).The median vein of the forearm and the median cubital vein are other veins which often have an adequate size for US-guided cannulation (Fig. 3).

### Key concept 2: Select patent veins

A fully patent vein is sine qua non for cannulation. This is demonstrated by applying slight compression forces over the skin with the transducer and observing veins that collapse easily (Additional file 2: Video S2). In contrast, a thrombosed vein is partially or totally non-compressible, is filled with thrombotic material (Additional file 3: Video S3) and thus is discarded for cannulation. After applying a tourniquet, a stagnant blood flow may be observed within the vein lumen in two-dimensional imaging, and this should not be confused with a thrombus (Additional file 4: Video S4). Distal compression aids in clearing this stagnant blood from the vein and ruling out a thrombus when vein patency is in doubt. Since peripheral veins have low blood flow velocity, spontaneous signal may not be observed in color Doppler. In these cases, distal compression allows to squeeze the blood from the vein, elevate blood flow velocity, thus aiding in the demonstration of flow in patent veins.

### Key concept 3: Determine vein size–catheter size

**Fig. 4:**
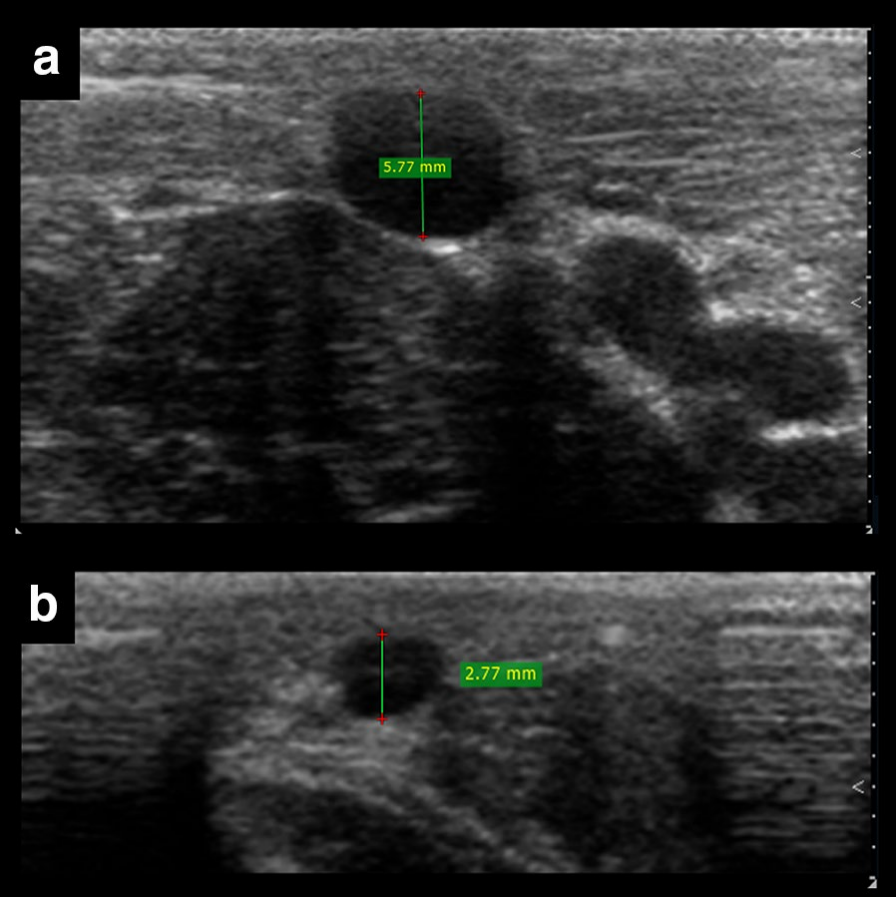
Two different anteroposterior diameters of the vein for US-guided cannulation. The vein in **a** has an excellent diameter for cannulation (> 4 mm), in contrast with the smaller vein in **b**

An optimal vein size is required to improve cannulation, and the size recommended in the literature is at least 4 mm in anteroposterior (AP) diameter [9] (Fig. 4). This suggested vein diameter, although important, should not be used in practice in a strict manner, since smaller veins can still be cannulated successfully, as seen in some studies [4, 6]. The vein size fulfills not only an established role in cannulation success (i.e., large veins are easily observed, as well as the needle within the vessel), but also aids in guiding catheter selection. As a rule of thumb, AP diameter indicates the upper limit of the external diameter of the catheter which can be used, considering that up to one-third of the vein lumen should be occupied by the catheter [10]. Thus, for example, a 4-Fr catheter (with an external diameter of 1.3 mm) is the maximum size for a 4-mm vein.

### Key concept 4: Determine vein depth–insertion angle–catheter length

**Fig. 5:**
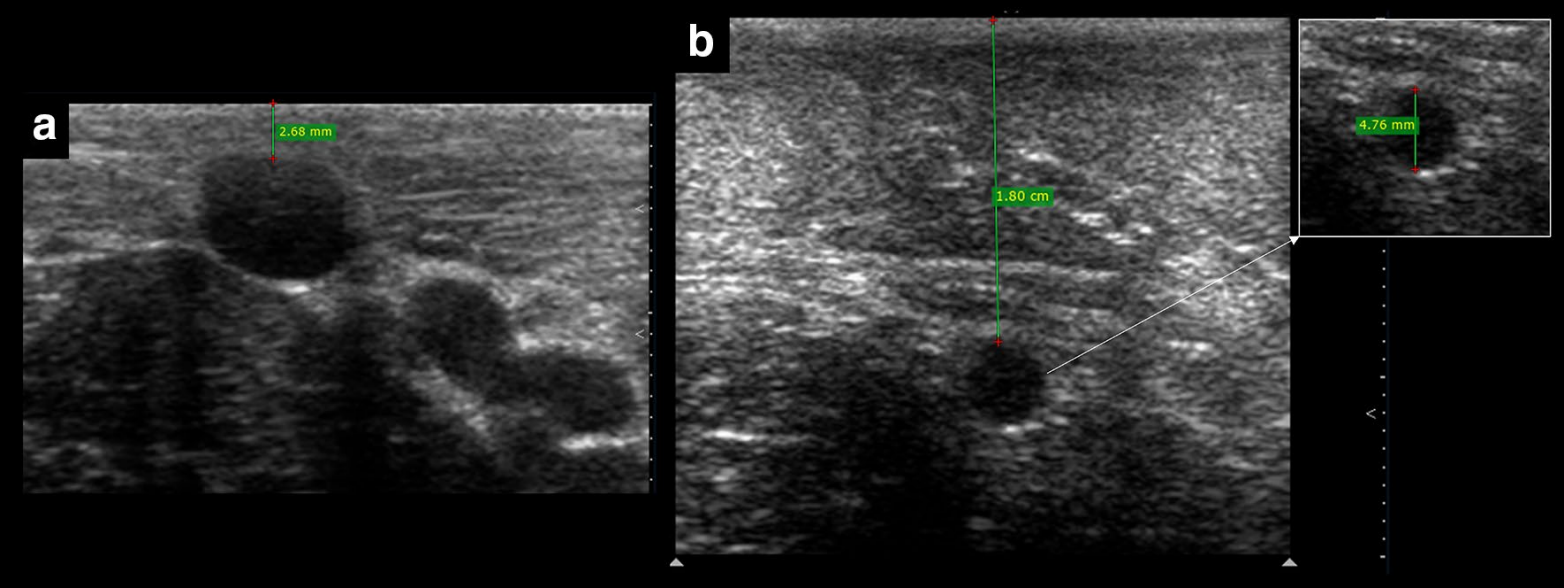
Distance from skin to vein for US-guided cannulation. In **a**, it is < 12 mm (ideal), while in **b** is > 16 mm. Since the vein in **b** has a diameter > 4 mm, it could still be cannulated using longer catheters than usual, for example, a midline catheter

**Fig. 6:**
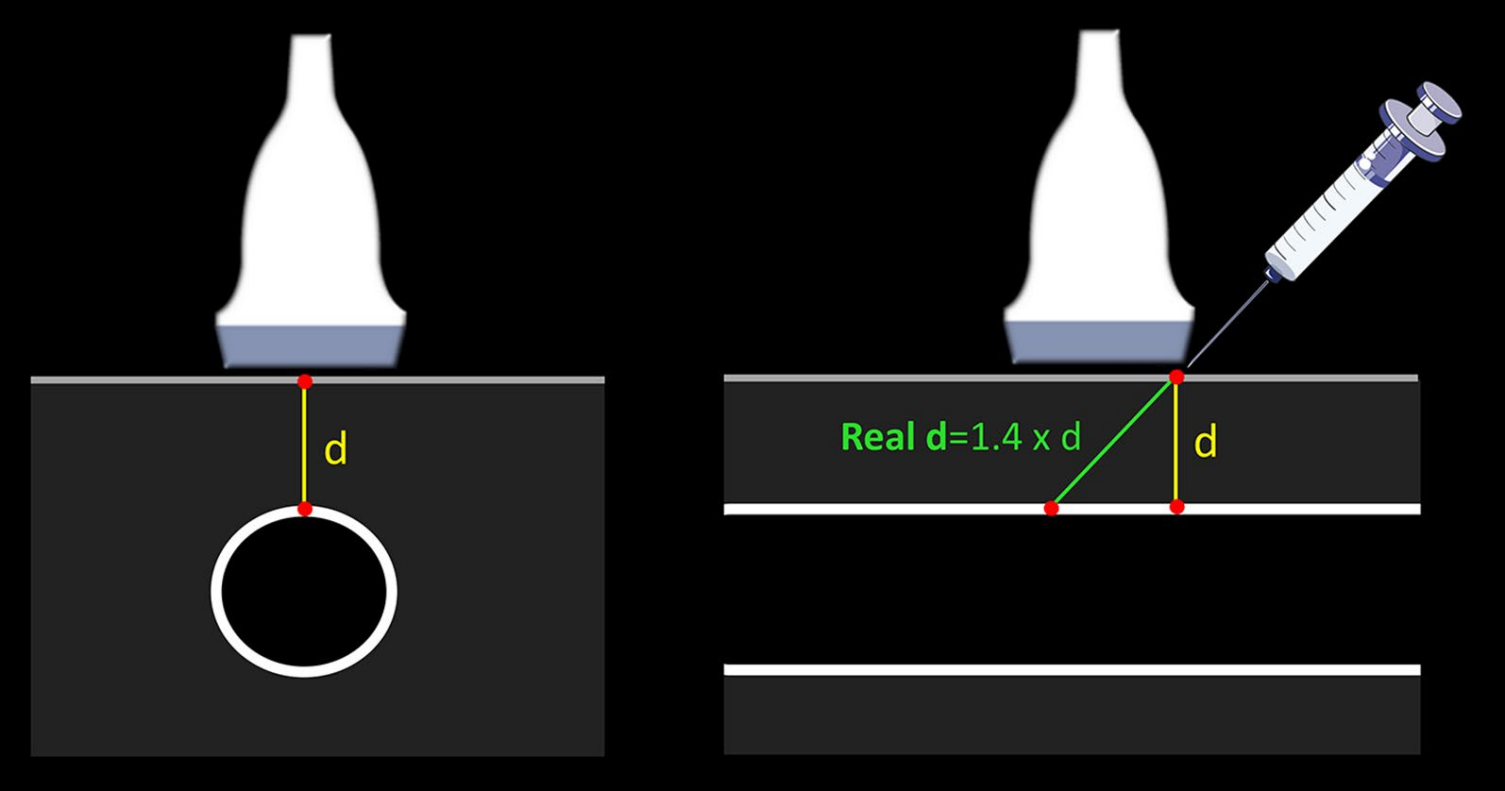
Explaining the distance from skin to vessel in ultrasound-guided cannulation. While this distance (*d*) is estimated in the short axis, the real distance to reach the vein depends on the insertion angle. Assuming a 45° insertion angle, this real distance is equal to *d* multiplied by 1.4. Of note, the real distance decreases with sloped insertions, and increases using shallower insertions

**Fig. 7:**
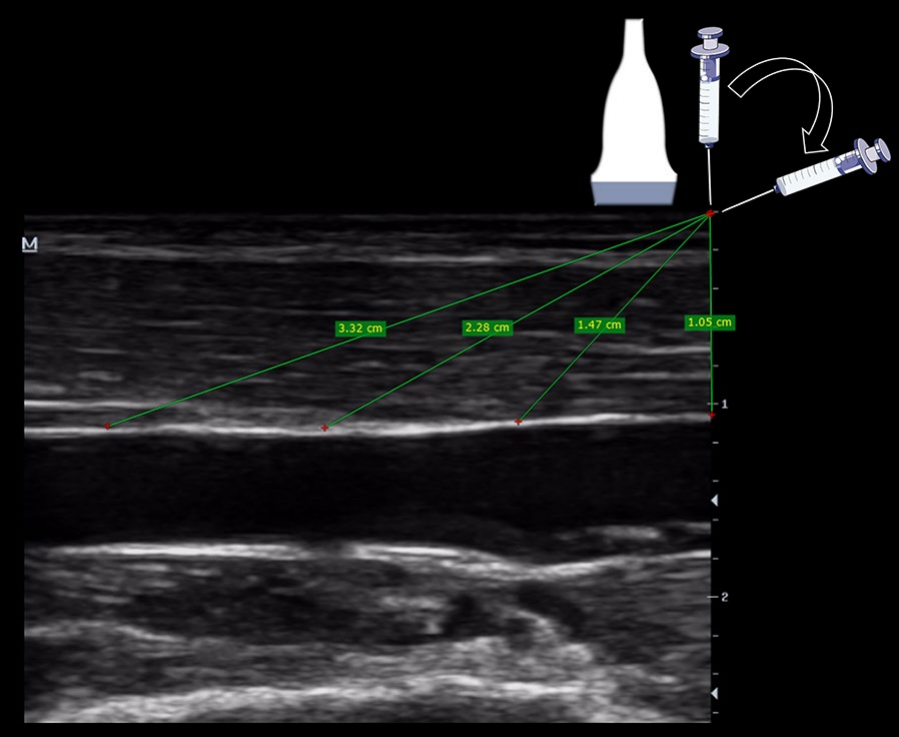
Real distance from skin to vein measured directly in the long axis. As shown, shallowest insertions determine a longest pathway to reach the vein, resulting in a large proportion of the catheter dwelling outside the vein and ultimately leading to catheter failure. In contrast, sloped insertions lead to shortening the distance to reach the vein, and aid in increasing the proportion of the catheter dwelling in the vein lumen

**Fig. 8:**
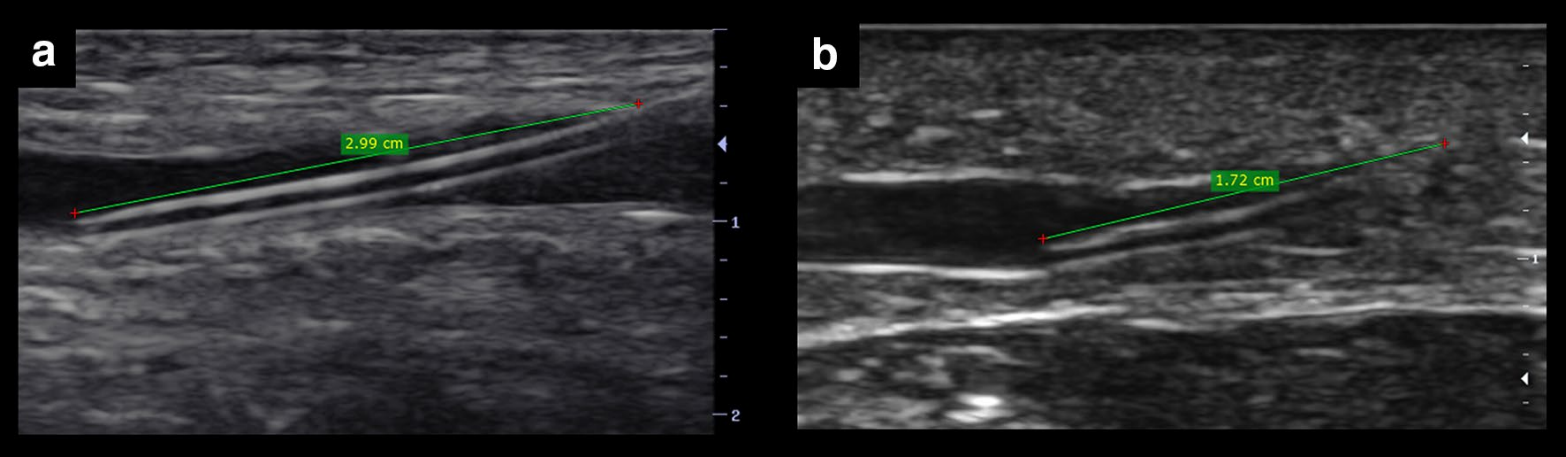
Amount of catheter residing in the vein. In **a.** the catheter dwelling in the vein is > 2.75 cm, in contrast with **b.** To achieve at least 2.75 cm of the catheter dwelling in the vein, several strategies can be used, for example, selecting veins at the lowest depth possible, using sloped insertion angles, and inserting catheters which are longer than usual, such as ultra-long peripheral catheters or midline catheters

As mentioned before, apart from avoiding the injury of the artery or nerve, practitioners should select superficial veins to guarantee a short pathway to cannulate the vein. The maximum suggested distance from skin to vein is < 16 mm [9, 11], while < 12 mm can be considered ideal [1] (Fig. 5). This distance supposes a 90° needle insertion related to the vessel, and thus, the real or “corrected” distance of the needle traveling to reach the vein can be approached performing Pythagorean assumptions, which are entirely true for a needle insertion angle of 45°. This distance is equal to 1.4 multiplied by the vertical distance (Fig. 6). For example, a vertical distance of 12 mm equals 16.8 mm using a 45° insertion. However, in practice, this length varies with the use of shallower (increased distance) or more sloped (decreased distance) needle insertions. Without the need to make calculations, practitioners can directly get this distance in the long axis, offering a big picture regarding the real distance to reach the vein when using different angles of insertion (Fig. 7). Of note, knowing this distance is of paramount importance to minimize PCF, given that a large proportion of the catheter must dwell in the vein [1, 6], and thus, a large distance to vein will result in a large proportion of the catheter outside the vein using standard-length catheters (SLC). A recent investigation using SLC showed that when < 30% (or one-third) of the catheter resides in the vein, all catheters failed. On the other hand, when > 65% (or two-thirds) of the catheter resides in the vein, none of the catheters failed. When 30–64% of the catheter was in the vein, 32.4% of intravenous catheters were lost [6]. More recently, comparing standard 4.78-cm-long catheters versus 6.35-cm-long catheters, Bahl et al. showed a significantly increased catheter survival when > 2.75 cm of the catheter resided in the vessel [12] (Fig. 8). This is coherent with the previous study, since 2.75 cm is closest to the 65% of an SLC. Thus, as a rule of thumb, achieving at least 2.75 cm of the catheter dwelling in the vein should be the cut-off used to mitigate catheter failure. This means, for example, that for an SLC of 4.78 cm, the “real” distance to reach the vein must be lower than 2 cm. To achieve this, several strategies can be used, for example, selecting vessels at the lowest possible depth, using sloped insertion angles, and inserting catheters which are longer than usual, such as ultra-long peripheral catheters (ULPC, 18-20G, 6.35 cm in length) and midline catheters (8–20 cm in length) [1, 8, 12, 13]. Using catheters which are longer than the standard size aids in minimizing PFC, allowing operators to use shallower insertion angles to improve needle visualization and also to select veins which are even deeper than 16 mm. Advantages of ULPC over midline are its low costs and the fact that they do not require advanced skills such as managing the Seldinger technique, so they can be inserted by nurses or technicians.

### Key concept 5: Select the cannulation technique: out-of-plane or in-plane technique

Practitioners should remember that each technique has advantages and disadvantages and thus learning and using both techniques are encouraged (Additional file 5: Video S5 and Additional file 6: Video S6), since they can select one or the other based on the situation [14, 15]. In a recent systematic review and meta-analysis, greater success has been shown with the out-of-plane technique compared to the in-plane technique [16]. However, for the out-of-plane technique, the visualization of the needle tip is an important limitation, having shown a higher rate of posterior wall perforations, compared to in-plane technique, which shows a clear delineation of the needle shaft and needle tip as it is advanced from superficial tissues into the vein [17, 18]. Using the “walk-down” maneuver (i.e., “follow the tip technique”) improves visualization of the needle tip when using out-of-plane insertions [14] and should be considered for using in practice. Side-lobe artifact is common when performing the in-plane technique, which simulates that the needle is inserted into the vein lumen, when is in fact close to it [14]. The learning curve for the in-plane technique seems to be longer compared to the out-of-plane technique [15].

### Key concept 6: Demonstrate the catheter is in the vein lumen and perform a saline flush test

**Fig. 9:**
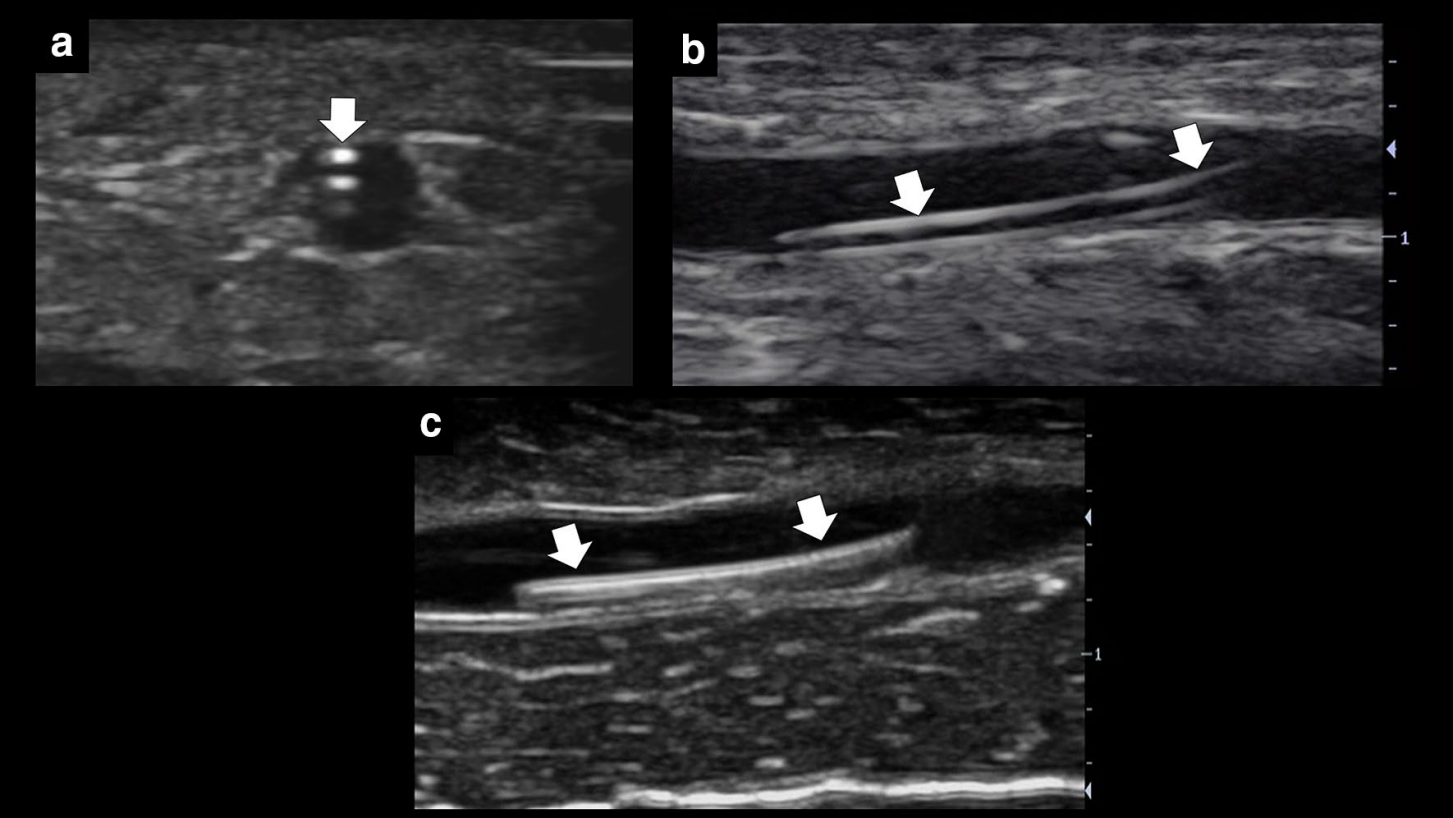
**a**, **b** Demonstration of the catheter (arrows) entering the vein lumen in short (**a**) and long axis (**b**); **c** demonstration of the guidewire entering the lumen vein (arrows) in the long axis, when using a midline catheter

After cannulating the vein, it is useful to check if the catheter is in the vein lumen, since is not infrequent that the infused solution passes easily to the subcutaneous tissue without any warning signs, thus delaying the institution of intravenous therapies. The catheter is observed as two parallel hyperechoic lines in the short, the long or both axes (Fig. 9a, b). In midline catheter insertions, the guidewire, seen as a hyperechoic linear structure, should be demonstrated before inserting the catheter (Fig. 9c). Finally, a saline flush test may be performed through the catheter, observing bubbles in the lumen vein in correctly positioned catheters (Additional file 7: Video S7); color Doppler can be used for this purpose as well [19].

## Conclusions

Practitioners should consider several issues when inserting intravenous peripheral catheters under ultrasound guidance, aiming to improve success rate, avoid complications and lengthen the survival of the catheter. Based on available data and everyday practice, all indicate that catheters longer than standard size are needed for US-guided peripheral venous cannulation, with the purpose of minimizing premature catheter failure. This is a call for attention to catheter manufacturers, since a more affordable solution at hand is expected from them shortly.

## Supplementary information

**Additional file 1: Video S1.** Real-time cross-section two-dimensional ultrasound imaging of the upper limb showing the anatomy of the peripheral veins and its relation to the fascia. Sv: superficial vein; Dv: deep vein; A: artery; N: nerve. As shown, superficial veins are epifascial, while deep veins are subfascial, and are accompanied by an artery and a nerve (in this case the deep veins are the paired brachial veins, the brachial artery and the median nerve).

**Additional file 2: Video S2.** Real-time two-dimensional ultrasound imaging showing patency of the peripheral veins, which are fully compressible.

**Additional file 3: Video S3.** Real-time two-dimensional and duplex ultrasound imaging showing a thrombosed superficial vein, which is non-compressible and filled by thrombotic material.

**Additional file 4: Video S4.** Real-time two-dimensional ultrasound imaging in long axis showing stagnant blood within the vein lumen after applying a tourniquet.

**Additional file 5: Video S5.** Real-time imaging showing the out-of-plane technique for ultrasound-guided vascular cannulation. The most relevant pros and cons of this technique are highlighted.

**Additional file 6: Video S6.** Real-time imaging showing the in-plane technique for ultrasound-guided vascular cannulation. The most relevant pros and cons of this technique are highlighted.

**Additional file 7: Video S7.** Saline flush test. Real-time two-dimensional ultrasound imaging in long axis showing bubbles appearing in the vein lumen immediately after flushing saline through the catheter, indicating a correctly positioned catheter.

## Data Availability

Not applicable.

## Abbreviations

DIVA: difficult intravenous access
PCF: premature catheter failure
US: ultrasound
SLC: standard-length catheters
ULPC: ultra-long peripheral catheters
